# Active surveillance for adverse events of influenza vaccine safety in elderly cancer patients using self-controlled tree-temporal scan statistic analysis

**DOI:** 10.1038/s41598-023-40091-y

**Published:** 2023-08-16

**Authors:** Na-Young Jeong, Chung-Jong Kim, Sang Min Park, Ye-Jee Kim, Joongyub Lee, Nam-Kyong Choi

**Affiliations:** 1 Department of Health Convergence, College of Science & Industry Convergence, Ewha Womans University, Seoul, Korea; 2 Department of Internal Medicine, Ewha Womans University Seoul Hospital, Seoul, Korea; 3 Department of Family Medicine, Seoul National University Hospital, Seoul, Korea; 4 Department of Biomedical Sciences, Seoul National University Graduate School, Seoul National University College of Medicine, Seoul, Korea; 5 Department of Clinical Epidemiology and Biostatistics, Asan Medical Center, University of Ulsan College of Medicine, Seoul, Korea; 6 Department of Preventive Medicine, Seoul National University College of Medicine, Seoul, Korea; 7 Graduate School of Industrial Pharmaceutical Science, College of Pharmacy, Ewha Womans University, Seoul, Korea

**Keywords:** Epidemiology, Epidemiology

## Abstract

Both cancer patients and the elderly are at high risk of developing flu complications, so influenza vaccination is recommended. We aimed to evaluate potential adverse events (AEs) following influenza vaccination in elderly cancer patients using the self-controlled tree-temporal scan statistic method. From a large linked database of Korea Disease Control and Prevention Agency vaccination data and the National Health Insurance Service claims data, we identified cancer patients aged over 65 who received flu vaccines during the 2016/2017 and 2017/2018 seasons. We included all the outcomes occurring on 1–84 days post-vaccination and evaluated all temporal risk windows, which started 1–28 days and ended 2–42 days. Patients who were diagnosed with the same disease during a year prior to vaccination were excluded. We used the hierarchy of ICD-10 to identify statistically significant clustering. This study included 431,276 doses of flu vaccine. We detected signals for 1 set: other dorsopathies on 1–15 days (attributable risk 16.5 per 100,000, P = 0.017). Dorsopathy is a known AE of influenza vaccine. No statistically significant clusters were found when analyzed by flu season. Therefore, influenza vaccination is more recommended for elderly patients with cancer and weakened immune systems.

## Introduction

Influenza is a potential cause of morbidity and mortality worldwide. The estimated overall rate of influenza-associated respiratory deaths each year is 4.0–8.8 per 100,000 individuals for all ages and 51.3–99.4 among individuals aged over 75 years^1^. Among them, immunocompromised patients, such as cancer patients, have a higher risk of death. They are more vulnerable to complications from influenza because factors related to immunosuppression affect the reaction to viral infection^2^. Previous studies showed that hospitalization for influenza was 5–10 times higher in cancer patients than in the general population, with a mortality rate of 9%^3^. To prevent influenza, influenza vaccination is widely recommended in the general population, especially for immunocompromised people, including cancer patients. In the United States, the Centers for Disease Control and Prevention (CDC) recommends influenza vaccination for cancer patients receiving chemotherapy or radiation treatment^4^. Similarly, publications released by the Korea Disease Control and Prevention Agency (KDCA) stipulate that cancer patients and immunocompromised patients are included in the priority targets for flu vaccination^5^.

Among cancer patients, the elderly have a particularly higher risk of influenza than other populations due to immunosenescence; thus, influenza vaccination for elderly cancer patients is strongly recommended. In Korea, KDCA has provided inactivated influenza vaccines free of charge every year to the elderly (aged 65 and over), children (aged 6 months through 12 years old), and pregnant women as part of the national immunization program (NIP)^6^, which shot up the influenza vaccination rate among the elderly to 86% as of 2019^7^. Considering the results of previous studies showing that the influenza vaccination rates of cancer patients and non-cancer elderly populations were similar^8^, the coverage of elderly cancer patients is expected to be very high. However, there have been few safety studies on influenza vaccination in elderly cancer patients in real-world settings. Most previous studies have demonstrated serological efficacy in patients treated with chemotherapy^9–11^, but the evidence for safety was relatively small. Although most studies related to the safety showed no significant difference in safety results in cancer patients vaccinated with influenza compared to the control group^10,12^, some patients with severe immune-related AEs such as encephalitis and pneumonia were observed^11^.

Since little is known about the safety of influenza vaccines in cancer patients from clinical trials or observational studies, it is important to identify potential adverse events (AEs) through post-marketing studies. Among the data mining methods for vaccine and drug safety surveillance, tree-based scan statistics has been recently introduced and used to detect safety signals without pre-specifying outcomes of concern^13,14^. In particular, tree-temporal scan statistics is a method evaluating whether any of a wide variety of health outcomes is temporally associated with the receipt of a specific drug^15^. Using this, it is possible to identify potential adverse events without pre-specifying the specific events or risk intervals of concern. Previous studies have demonstrated that the self-controlled tree-temporal scan statistic method is applicable to simultaneously evaluate a wide range of vaccine-adverse reactions^16–19^. Although rigorous epidemiological studies might be required to confirm the signals detected by this method, it helps identify previously unknown and unexpected safety issues.

Thus, the aim of the present study was to identify potential adverse events following influenza vaccination in elderly cancer patients by applying the self-controlled tree-temporal scan statistic method and to find unexpected and unknown adverse events.

## Material and methods

### Data sources

The study was performed based on the vaccination registration database from the KDCA and the health insurance claims database from the National Health Insurance Service (NHIS). In Korea, since vaccines are one of the non-covered medications under national health insurance, vaccination records for NIP vaccines can be identified through the computerized registration database of National Immunization Registry Integration System^20^. Vaccination by NIP for the entire population should be recorded electronically to reimburse the cost of vaccines^20^. The vaccination registration database includes information such as vaccination date, injection site, vaccination clinic, and type of vaccine. To identify the medical utilization information, we used the health insurance claims data generated in the process of managing the national healthcare insurance program in Korea. Since the National Health Insurance Act was enacted in 1999, the Health Insurance and Review Assessment (HIRA) has reviewed medical fees for reimbursement decisions and the NHIS reimburses healthcare services based on the assessment results from HIRA^21,22^. From the reimbursement process, the NHIS constructed a health insurance claims database that covered the entire population of 50 million and provided the data to researchers for study. The NHIS database comprises information on sociodemographic characteristics, diagnostic information, drug prescription information, and procedure information.

We used the linked databases of the national immunization registry data and the national health insurance claims data between 2015 and 2018. To link vaccination registration data and NHIS claims data, we requested that the KDCA deliver vaccination registration data to the NHIS. The NHIS linked two databases using resident registration numbers and provided us with anonymized data.

### Study population and exposure

Cancer patients who received influenza vaccines during the 2016/2017 and 2017/2018 seasons and who were aged 65 years or older on the date of influenza vaccination were included in the eligible study population. We excluded patients who were less than 65 years old because they were not covered under NIP for influenza vaccination. Cancer patients were identified as those diagnosed with cancer from at least one inpatient setting or from at least three outpatient settings for a year prior to vaccination. We used the International Classification of Diseases Tenth Revision (ICD-10) code (C00-C97: Malignant neoplasms) and rare and intractable disease registration program code for cancer (V027, V193, V194: cancer-related codes for exempted calculation of health insurance) simultaneously to minimize misclassification of diagnosis. Since only the year of birth could be identified in the database, the age of the subjects was calculated by assuming the date of birth as January 1.

The two flu seasons used in this study were defined as follows: the 2016/2017 season, corresponding to the period from September 1, 2016, to April 30, 2017, and the 2017/2018 season, corresponding to the period from September 1, 2017, to April 30, 2018, by referring to previous studies^23,24^. For those who received more than one dose per season, only the first dose per season was included in the analysis because the risk and control intervals for each dose could overlap. If an individual was vaccinated during two flu seasons, each vaccination was considered independent.

### Hierarchical diagnosis tree

Tree‐temporal scan data mining method is conducted based on a predefined tree structure. We used a hierarchical tree structure defined by ICD-10 code to identify outcomes. ICD-10 codes have a hierarchical structure with five levels, from the broadest at the top to the most specific diagnosis code at the bottom (Supplement Fig. 1). The first and broadest level contains 21 sections classified by functional apparatus^25^.

Some diagnosis codes indicating the following conditions were excluded from the tree (Supplement Table 1): diagnosis used in the definition of the study subject such as neoplasms, outcomes in the periods of pregnancy, perinatal, childbirth, infantile, or juvenile; congenital or hereditary conditions; diagnosis codes related to external causes of morbidity and mortality; and diagnosis codes related to factors influencing health status and contact with health services. Finally, the resulting pruned tree contained 5,866 ICD-10 codes containing 14 first levels.

### Incident outcomes

The outcomes of interest were focused on ‘incident’ diagnoses in the outpatient, inpatient, or emergency department setting during the follow-up period. To examine only incident diagnoses after vaccination, the diagnoses observed with the same first 3 letters of ICD-10 code during a year prior to vaccination were excluded. This was to avoid overestimating the occurrence or redundant counting of similar diseases. We did not look for clustering in the first (broadest) and second levels of the tree. Each patient was allowed to contribute to multiple outcomes during the follow-up period unless their diagnoses belonged to the same three levels of the tree.

### Risk and control windows

**Figure 1:**
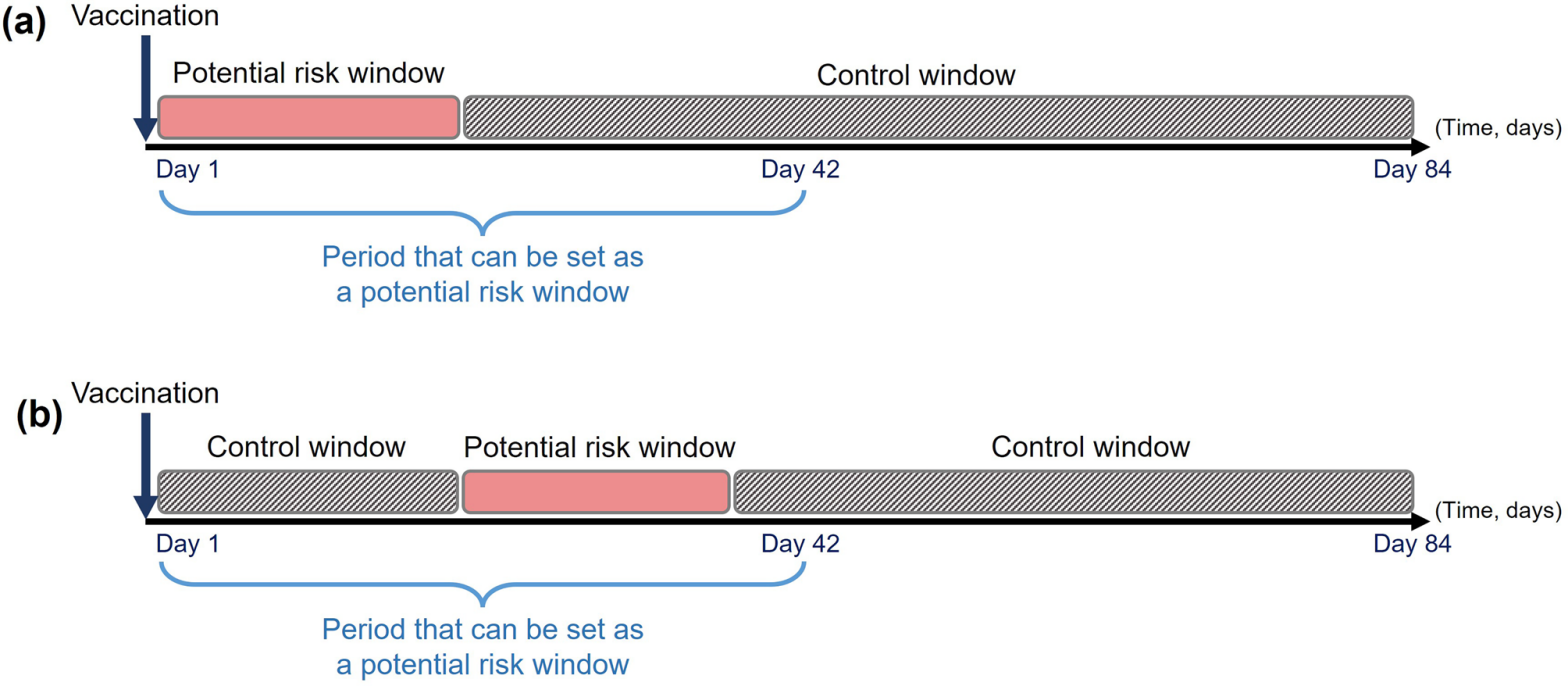
Illustration of risk and control windows for self-controlled tree-temporal tree-based scan statistic analysis. Examples of potential risk windows evaluated with their control period(s), assuming that 84 days of complete follow-up exists for the patient. The individuals participating in the study have at least one risk window and one control window, respectively. (**a**) A potential risk window that starts on day 1 following influenza vaccination. The corresponding control period begins the day after the end of the potential risk window and extends to day 84. (**b**) A potential risk window located somewhere in the middle of the follow-up period. The corresponding control period consists of the sections of the follow-up period that are not in the potential risk window.

We included all the health outcomes occurring on 1–84 days (12 weeks) following the first influenza vaccination per flu season in the analysis. The follow-up period was selected to include potential adverse events, taking into account the time from occurrence to diagnosis and coding. The day of vaccination was not included in the follow-up period on account of the possibility of a preventive-care visit or a health-care visit due to other health concerns. The length of temporal risk windows was between 2 and 42 days, which starts between 1 and 28 days after vaccination and ends between 2 and 42 days after vaccination (Fig. 1). The control window was defined as the remaining days within the follow‐up period but outside the risk window. To avoid lowering power due to analysis of implausible risk intervals (e.g. short risk windows after a long time following vaccination), the length of the minimum temporal risk window was limited to be atleast 20% of the distance between the time zero and the end of the risk window.

### Tree‑temporal scan statistic (statistical analysis)

Tree-temporal scan statistic is a method used to evaluate a broad range of diagnosis codes for various clinical outcomes and groups of related outcomes. It also considers multiple potential risk windows simultaneously. While adjusting for multiple tests, the number of cases within the risk window is compared with the number of cases within the control window that would be expected by chance. This comparison assumes that the incidences of the cases were randomly and uniformly distributed over time. All the statistically unusual clusterings of cases within a large hierarchy are detected. Tree-temporal scan statistic with a self-controlled design makes within-person comparisons among time periods, therefore all time-invariant confounders were adjusted. We only calculated the number of events within the risk or control window for vaccinated individuals. The cases of any event among unvaccinated people are not measured. The null hypothesis is that there is no unusual temporal clustering of events on any leaf or branch across the study time period, while the alternative hypothesis is that there is at least one cut on a leaf or a branch having a temporal clustering of events for some time windows. This method is useful when it is difficult to set a risk window due to a lack of safety information because it is not necessary to set a well-defined risk section in advance. By using the method, we can find out whether the influenza vaccine causes a very specific reaction, such as acute disseminated encephalitis (lower level) or a broader group of related AEs, such as inflammatory diseases of the central nervous system (higher level).

Poisson generalized log likelihood ratios (LLRs) test statistic for each tree node and time interval was calculated. We conditioned on the number of cases observed in each node of the tree as well as the total number of events occurring on each day during the follow-up period. The number of Monte Carlo replications selected for this analysis was 9999 to obtain a *p*-value for the detected temporal cluster in a situation where the distribution of the test statistic was not known^16^. This adjusts for the multiple testing by comparing the test statistics generated from the real dataset with test statistics generated from all other random datasets. We detected temporal clusters with *p* ≤ 0.05 as signals. Attributable risk was calculated by dividing the excess number of cases by the total number of cancer patients exposed to flu vaccine^26^.

The analysis was conducted using SAS enterprise guide version 7.1 (SAS Inc., Cary, NC, USA) and TreeScan software v2.1 (https://www.treescan.org).

### Ethics statement

This study was approved by the Institutional Review Boards of Ewha Womans University (ewha-202210-0005-01) and received a waiver of informed consent because we used the linked databases containing anonymized data that cannot allow patients’ identification. All methods were performed in accordance with the relevant guidelines and regulations.

## Results

**Figure 2:**
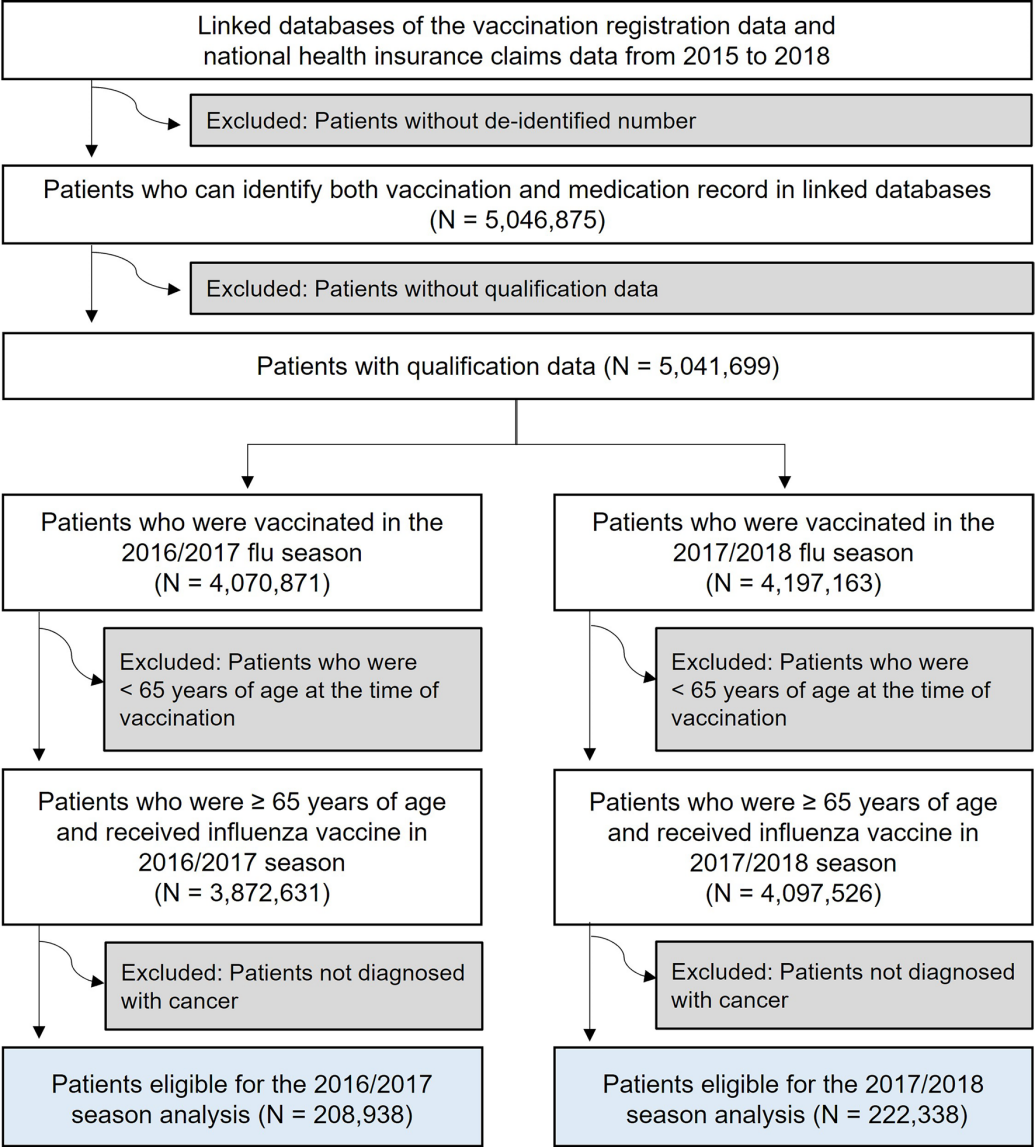
Flow chart for the inclusion of eligible patients.

During the study period, a total of 7,970,157 doses of influenza vaccine were received by the elderly (3,872,631 doses in the 2016/2017 season; 4,097,526 doses in the 2017/2018 season). Among these, 431,276 doses administered to cancer patients were included in the analysis (208,938 doses in the 2016/2017 season; 222,338 doses in the 2017/2018 season). The flow chart is presented in Fig. 2. Note that most vaccinations occurred in September (81,517; 18.9%) or October (326,725; 75.8%), and the number of men (260,205; 60.3%) was higher than that of women (171,071; 39.7%) (Table 1).

**Table 1:** Descriptive characteristics related to first doses of influenza vaccine in the elderly cancer patients during 2016/2017 and 2017/2018 flu seasons.

Characteristics	2016/2017 season	2017/2018 season
Total	208,938 (100%)	222,338 (100%)
Age		
Mean ± SD	74.1 ± 6.1	74.3 ± 6.2
Median (IQR)	74 (69–78)	74 (69–78)
65–74	116,237 (55.6%)	118,541 (53.3%)
75–84	80,510 (38.5%)	89,531 (40.3%)
Over 85	12,191 (5.8%)	14,266 (6.4%)
Gender		
Female	83,749 (40.1%)	87,322 (39.3%)
Male	125,189 (59.9%)	135,016 (60.7%)
Month of vaccination		
September	560 (0.3%)	80,957 (36.4%)
October	199,868 (95.7%)	126,857 (57.1%)
November	7002 (3.4%)	12,801 (5.8%)
December	1267 (0.6%)	1211 (0.5%)
January	183 (0.1%)	449 (0.2%)
February	41 (0.0%)	54 (0.0%)
March	16 (0.0%)	5 (0.0%)
April	1 (0.0%)	4 (0.0%)
Vaccinated regions*		
Capital region	82,469 (39.5%)	88,405 (39.8%)
Metropolitan city	50,505 (24.2%)	54,154 (24.4%)
Others	75,959 (36.4%)	79,778 (35.9%)
Type of vaccinated institution		
Private health institution	177,020 (84.7%)	193,125 (86.9%)
Public health institution	31,918 (15.3%)	29,213 (13.1%)

*SD* standard deviation, *IQR* interquartile range.

*Only people whose vaccinated regions information was not missing in the database were shown in the table.

**Figure 3:**
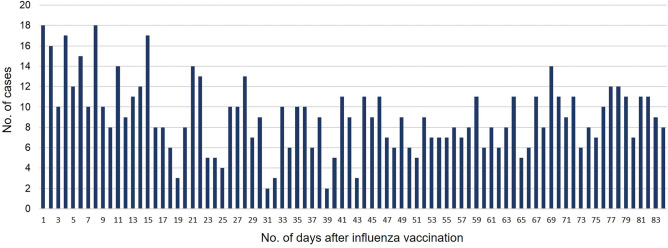
Temporal distribution of ‘other dorsopathies’ cases according to the number of days to diagnosis after influenza vaccination among elderly cancer patients.

Within the observation period, 667,323 incidence AEs associated with 1582 ICD-10 codes were identified. Table 2 lists all diagnoses included in the most likely cuts, meaning that the clusters of cases were least likely to occur by chance. Even though the most likely cuts have an excess rate, as shown in Table 2, there will invariably be some area with a rate higher than expected purely by chance alone under the null hypothesis^26^. There was only one diagnosis which presented statistical alerts (*p*** ≤ **0.05); ‘other dorsopathies’ on 1–15 days following vaccination. We found 197 cases in the risk window (days 1–15 after vaccination) and 563 cases in the control window (days 16–84 after vaccination). However, more specific codes on the lower levels in this branch were not detected as signals. The attributable risk of other dorsopathies per 100,000 influenza vaccinees was 16.5. The temporal distribution of the occurrence of the disease from the date of influenza vaccination is presented in Fig. 3. Other dorsopathies showed a higher distribution of the number of patients in the early stages of vaccination, and the number of cases varied from 1 to 18 cases per day.

**Table 2:** List of adverse events from tree-temporal scan statistical analysis of influenza vaccination in elderly cancer patients, from the 2016/2017 through 2017/2018 flu seasons.

Node code	Text description	Risk window following vaccination	Observed number of cases in risk window	Attributable risk per 100,000 doses	*p*-value*
B35-B49	Mycoses	25–34 days	45	5.1	0.994
G21.9	Secondary parkinsonism, unspecified	3–4 days	2	0.5	> 0.999
G43.8	Other migraine	24–30 days	4	0.9	> 0.999
H43-H45	Disorders of vitreous body and globe	24–30 days	13	2.2	0.997
H43	Disorders of vitreous body	24–30 days	13	2.3	0.994
H49-H52	Disorders of ocular muscles, binocular movement, accommodation and refraction	16–20 days	26	3.4	0.999
H52	Disorders of refraction and accommodation	16–20 days	26	3.4	0.995
H52.2	Astigmatism	17–20 days	14	2.3	0.967
H52.4	Presbyopia	5–6 days	4	0.9	0.850
L20-L30	Dermatitis and eczema	6–40 days	473	26.8	> 0.999
M20-M25	Other joint disorders	14–18 days	30	3.7	0.997
M22	Disorders of patella	22–27 days	4	0.9	0.972
M25	Other joint disorder, not elsewhere classified	14–18 days	26	3.4	0.999
M50-M54	Other dorsopathies	1–15 days	197	16.5	0.017
M51	Thoracic, thoracolumbar, and lumbosacral intervertebral disc disorders	8–15 days	27	3.6	0.998
N93	Other abnormal uterine and vaginal bleeding	11–20 days	11	2.1	0.998
N93.9	Abnormal uterine and vaginal bleeding, unspecified	12–20 days	8	1.6	> 0.999
R22	Localized swelling, mass and lump of skin and subcutaneous tissue	1–2 days	6	1.2	> 0.999
R59	Enlarged lymph nodes	7–9 days	4	0.9	0.995
R59.1	Generalized enlarged lymph nodes	7–9 days	3	0.7	0.999

*p-value was estimated tree-temporal scan statistical analysis with Monte-Carlo permutation-based methods that adjust for the multiple comparisons made among many branches and the tree.

According to the subgroup analysis by flu season, there was no statistically significant signal. The most likely cuts included ‘other dorsopathies’, but with no statistical significance; 61 cases in days 1–8 following vaccination in the 2016/17 season (*P* = 0.953) and 76 cases in days 1–11 following vaccination in 2017/18 season (*P* = 0.986).

## Discussion

This study delineated whether there were potentially increased adverse events following influenza vaccination in elderly cancer patients based on the nationwide Korean population. With a self-controlled tree-temporal scan statistical method, we found one signal of a potential adverse event in the ICD-10 coding system within 15 days after vaccination. Using this method, we could not find evidence that influenza vaccine was associated with any adverse events in the first 6 weeks after vaccination, other than ‘other dorsopathies’.

Other dorsopathies include a wide range of disease areas, including cervical disc disorders, other intervertebral disc disorders, and dorsalgia. Although in this study, among the patients diagnosed with ‘other dorsopathies (M50-M54)’, patients diagnosed with ‘dorsalgia (M54)’ during the observation period contributed a lot (527 patients), but they did not correspond to a likely cut. Since no significant cuts were found in other diagnosis codes related to dorsopathies, it is hard to explain that influenza vaccination is related to a risk of a specific type of backache in cancer patients.

Dorsopathy, including back pain, is one of the adverse events of influenza vaccines specified in Micromedex, with cases reported from 1 to 21 days following vaccination in randomized clinical trials. The actual incidence rate has not been precisely known. However, there were few case reports of severe neurological diseases in patients who have experienced back pain symptoms after influenza vaccination. According to case reports, patients were diagnosed with neuromyelitis optica spectrum disorder (NMOSD)^27^, acute fulminant myocarditis^28^, or acute disseminated encephalomyelitis^29^ a few days after experiencing upper or lower back pain after vaccination with inactivated quadrivalent influenza vaccination. Therefore, it is necessary to conduct research on the risk of other serious neurological diseases accompanied by back pain. Additionally, further pharmacoepidemiological studies are needed to evaluate whether the risk of developing dorsopathies is particularly increased by influenza vaccination in cancer patients.

The current study makes a unique contribution to the safety profile of influenza vaccination for elderly cancer patients in several aspects. First, the self-controlled tree-temporal scan statistic method has been identified as one of the good signal-detection methods to generate hypotheses in previous studies. Unlike most vaccine safety studies, we did not limit the assessment to a few health outcomes. It is the first study to evaluate all possible adverse events following influenza vaccination in elderly cancer patients in Korea using this method. Second, since data on all elderly cancer patients in Korea were used, relatively rare adverse reactions were identified, and the results were highly representative. Even though the claims data were used, the validity of cancer diagnosis was relatively secured because the rare incurable disease registration code was used to define cancer patients.

Despite these strengths, some limitations still remain. First of all, we assessed potential adverse events occurring only within 6 weeks of vaccination. Therefore, we might have missed an increased risk of a true adverse event whose period of risk was beyond 6 weeks or whose risk was constant throughout the follow-up period. In addition, the fact that most of the influenza vaccines evaluated in this study are typically administered in the fall may cause time-varying confounding, which generates a false safety signal. Although it did not emerge in this study, in-depth and customized interpretation will be required when interpreting signals related to exposures with temporal trends, such as influenza vaccines. Moreover, the validity of the entire diagnostic code in health insurance data has not been evaluated. Although previous studies evaluating the validity in claims data in Korea showed high reliability for several diseases such as cancer, acute myocardial infraction, stroke, and inflammatory bowel disease^30–33^, attention needs to be paid to the interpretation of our findings that have screened all diagnostic codes using the tree-temporal scan statistic method. The last thing to note is that detected signals do not mean causality between influenza vaccination and adverse events. To examine causation and the exact time period of increased risk of adverse events in elderly cancer patients, further pharmacoepidemiological studies are needed.

In conclusion, we identified 1 known safety signal within 12 weeks after vaccination for elderly cancer patients using the self-controlled tree-temporal scan statistic method. Overall, our findings provide reassurance of the safety of influenza vaccine in the elderly cancer patient population. In addition to all cancer patients, additional research is needed on whether there is a difference in adverse reaction signals according to cancer types. Given that the influenza vaccine formulations change annually, continuously monitoring of the risk of adverse events during future flu seasons is necessary. Further studies are needed to validate safety signals detected and conduct rigorous future epidemiological studies that incorporate adjustment of confounding factors and employ outcome definitions based on validated algorithms.

### Supplementary Information

Supplementary Information.

## Data Availability

The datasets use and/or analysed during the current study are not publicly available. The data that support the findings of this study are available from the corresponding author, [N.K.C.], upon reasonable request.
