# Supplementary material for: Active surveillance for adverse events of influenza vaccine safety in elderly cancer patients using self-controlled tree-temporal scan statistic analysis
Source: Sci Rep. 2023 Aug 16;13:13346. doi: 10.1038/s41598-023-40091-y (PMC10432531; doi:10.1038/s41598-023-40091-y)
Supplement: Supplementary file 1 — Supplementary Information. [file 41598_2023_40091_MOESM1_ESM.pdf]

Excluded ICD-10 codes	Description
C00-D48	Neoplasms (cohort defining diagnoses)
O00-O99	Pregnancy, childbirth and the puerperium
P00-P96	Certain conditions originating in the perinatal period
Q00-Q99	Congenital malformations, deformations and chromosomal abnormalities
V01-Y98	External causes of morbidity and mortality
Z00-Z99	Factors influencing health status and contact with health services
U00-U99	Codes for special purposes
E550, F642, F840, F843, F80-89, F90-F98, G120, H260, L122, L211, L444, L704, M08, M09, M302, M330, M410, M411, M420, M91, M92, R62, R681, R95	Infantile/Childhood/Juvenile diseases
A50, D58, D640, D644, D66, D67, D680, D681, D682, D720, D740, D800, E00, E030, E031, E730, E800, E850, E851, E852, G11, G121, G60, G702, G712; H185, H312, H355, H710, I780, M140, M23.1, N07	Congenital/Inherited/Hereditary diseases

Supplementary Table 1. International Classification of Diseases, Tenth Revision, Coding System excluded from the tree

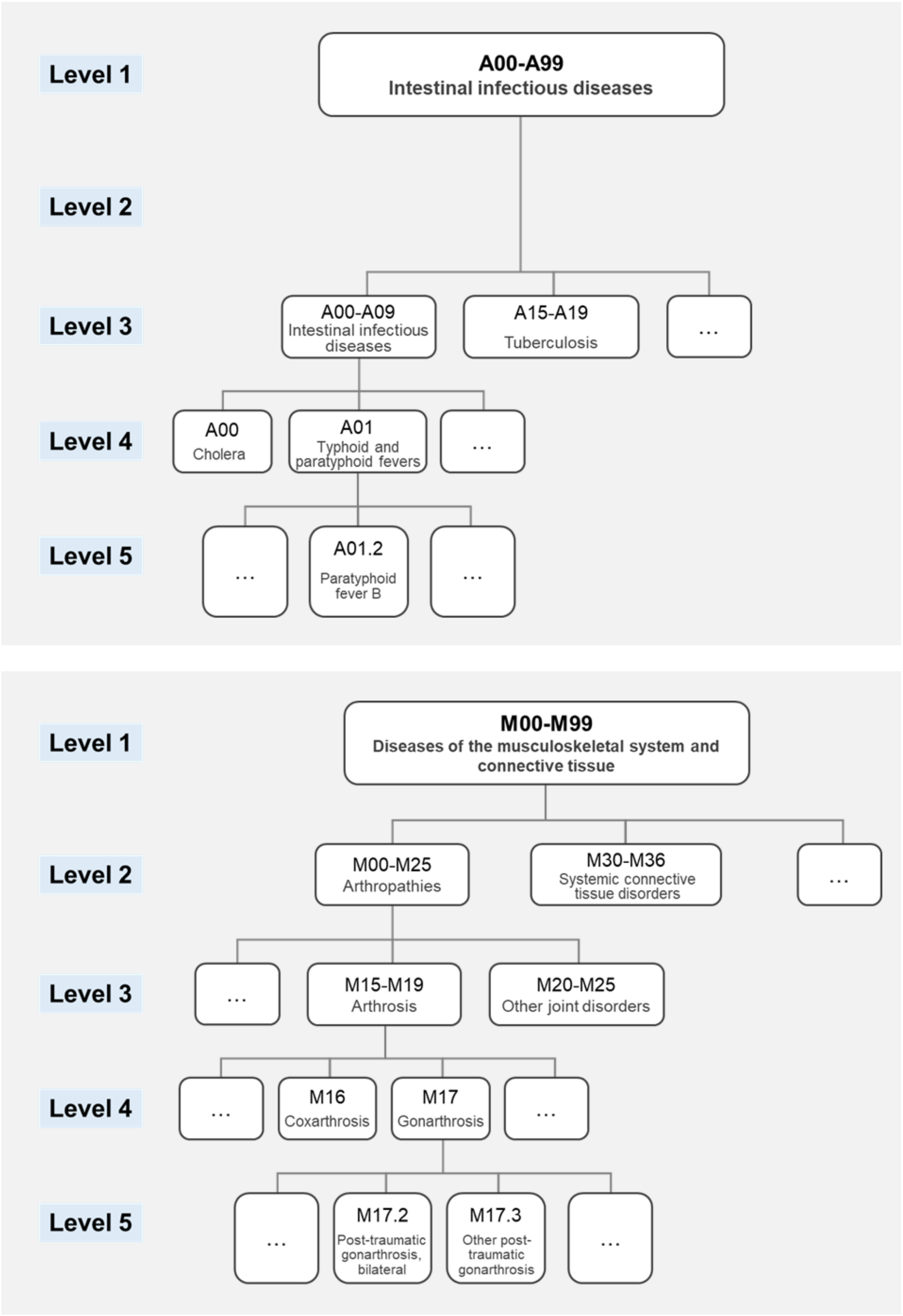

Supplementary Figure 1. Examples of hierarchical tree structure in the International Classification of Diseases, Tenth Revision, Coding System.
